# The association of blood angioregulatory microRNA levels with circulating endothelial cells and angiogenic proteins in patients receiving dacarbazine and interferon

**DOI:** 10.1186/1479-5876-10-241

**Published:** 2012-12-05

**Authors:** Pierre L Triozzi, Susan Achberger, Wayne Aldrich, Arun D Singh, Ronald Grane, Ernest C Borden

**Affiliations:** 1 Taussig Cancer Institute, Cleveland Clinic Foundation, 9500 Euclid Avenue, Cleveland, OH, 44195, USA; 2 Cole Eye Institute, Cleveland Clinic Foundation, Cleveland, OH, USA

**Keywords:** Biomarker, Tumor angiogenesis, Vascular endothelial growth factor, Basic fibroblast growth factors, Interleukin-8, Melanoma

## Abstract

**Background:**

Blood biomarkers are needed to monitor anti-angiogenic treatments for cancer. The association of blood levels of microRNAs (miRs) implicated in angiogenesis with circulating endothelial cells (CEC) and with angiogenic proteins was examined in patients administered drugs with anti-angiogenic activity.

**Methods:**

Blood was collected from patients with uveal melanoma enrolled on an adjuvant therapy trial in which they were treated sequentially with dacarbazine and interferon-alfa-2b. Plasma levels of nine angioregulatory miRs, miR-16, 20a, 106a, 125b, 126, 146a, 155, 199a, and 221, were determined by quantitative real time polymerase chain reaction; CEC, by semi-automated immunomagnetic; and plasma angiogenic proteins, by enzyme linked immunosorbent assays.

**Results:**

Levels of miR-199a were positively correlated and miR-106a negatively correlated with CEC pre-therapy. Decreases in miR-126 and miR-199a and increases in miR-16 and miR-106a were observed after interferon-alfa-2b, but not after dacarbazine. CEC also increased after treatment with interferon but not after treatment with dacarbazine. Levels of miRs did not correlate with levels of vascular endothelial growth factor, basic fibroblast growth factor, and interleukin-8. Angiogenic proteins also did not change significantly with treatment.

**Conclusions:**

Blood levels of specific angioregulatory miRs are associated with CEC, and changes in specific angioregulatory miRs parallel increases in CEC after treatment with interferon-alfa-2b. Blood levels of specific angioregulatory miRs are not associated with levels of angiogenic proteins. miRs warrant further evaluation as blood biomarkers of angiogenesis.

## Background

A number of drugs with anti-angiogenic effects are in common use to treat cancer, and a number are under investigation. Although many methods have been tested in preclinical and clinical studies, there are no established methods of serially monitoring patients receiving anti-angiogenic therapies. Several studies have focused on known protein mediators of angiogenic processes. Changes in blood levels of, *e*.*g*., vascular endothelial growth factor (VEGF), basic fibroblast growth factors (bFGF), and interleukin- (IL-) 8, have been observed in response to anti-angiogenic drugs. The results have been conflicting, due in part to the different clinical situations investigated. Their use may also be confounded by increases associated with tumor progression, and the practical utility of using drug-induced changes in angioregulatory proteins as blood biomarkers remains to be demonstrated [1].

Circulating endothelial cells (CEC) are mature endothelial cells that have detached from the vessel wall and are considered to indicate vascular damage. Several clinical trials have associated changes in CEC with outcome to anti-angiogenic treatments. Increases in CEC were associated with clinical benefit in studies in renal [2], pancreatic [3], and breast cancers [4] and gastrointestinal stromal tumor [5]. In patients with solid tumors randomized to anti-angiogenic drug combinations, CEC were lower in patients without clinical benefit; of note, levels of VEGF did not differ in these patients [6]. In contrast, in studies in colon cancer [7] and in glioblastoma [8], an increase in CEC was associated with a worse clinical outcome, and in a study in breast cancer, a decrease in CEC was associated with clinical response [9]. Still in others, including studies in colon [10] renal [11], and hepatocellular cancers [12], changes in CEC were not associated with clinical outcome. Furthermore, tumor progression is accompanied by increases in CEC [13].

Tumor angiogenesis has been shown to be regulated by microRNAs (miRs), small noncoding RNAs that bind to mRNAs, recruit a silencing complex, and block translation. miRs that can promote angiogenesis, including miR-126, 155, 199a, and miRs of the 17–92 complex, and miRs that can inhibit angiogenesis, including miR-16, 106a, 125b, and 221, have been identified. These miRs have been implicated in the regulation of a range of target genes involved in angiogenesis, such as those involved in response to hypoxia, production of angiogenic proteins/growth factors, and endothelial cell proliferation and migration [14]. miRs are very stable in blood because of incorporation in microparticles and exosomes. Because of sensitive detection methods and their low complexity, when compared to proteins and cells, blood miRs are under investigation as cancer biomarkers [15]. There is, however, little information regarding the effects of drugs with anti-angiogenic effects on circulating miRs.

With the overall objective of developing blood biomarkers of angiogenesis, we examined the associations of blood levels of miRs implicated in angiogenesis with those of angiogenic proteins and CEC in patients with primary uveal melanoma on systemic adjuvant therapy consisting of low-dose dacarbazine followed by interferon-alfa-2b, drugs with anti-angiogenic effects. Interferon-α has well-documented inhibitory effects on endothelium and angiogenic factors [16]. Dacarbazine, like temozolomide, is a prodrug of the alkylating agent 5-[3-methyltriazen-1-yl]imidazole-4-carboximide, which also has demonstrated inhibitory effects on endothelium and angiogenic factors [17]. Increases in CEC have been observed in clinical trials that included interferon-alfa-2b and temozolomide [18,19]. In addition, the patients we evaluated did not have clinically detectable cancer, which lessened the potential impact on cancer burden on the biomarkers tested. The sequential treatment program utilized, which improved disease-free survival in resected, high-risk cutaneous melanoma [20], also allowed for the comparison of an anti-angiogenic chemical and an anti-angiogenic cytokine within the same patient.

## Methods

### Patients and treatment

This study was approved by Cleveland Clinic Institutional Review Board and was in compliance with the Helsinki Declaration. All subjects provided written informed consent. The patients enrolled had a histocytologic diagnosis of melanoma of the iris, ciliary body and/or choroid; high-risk tumor cytogenetics (*i*.*e*., monosomy 3), adequate primary therapy (*e*.*g*., enucleation or brachytherapy); negative imaging to eliminate distant disease; performance status (ECOG) less than 2; and normal organ function. Patients had to be entered within 56 days of completing primary therapy. Dacarbazine was administered at 850 mg/m^2^ intravenously on day 1 and day 28. Interferon-alfa-2b was administered at 3 million units three times a week subcutaneously for 24 weeks beginning week 9. Patients were pre-medicated with 650 mg acetaminophen prior to interferon-alfa-2b. Blood for laboratory correlates was drawn and analyzed week 1, prior to starting dacarbazine in 21 patients. Blood was also drawn and analyzed in 12 patients at week 9, after dacarbazine and prior to starting interferon; weeks 17, 25, and 33, on interferon; and at 6 months after therapy was discontinued.

### Circulating miRs

Total RNA was isolated from plasma using the miRNeasy Mini Kit (Qiagen, Valencia, CA) according to the manufacturer’s instructions. Reverse transcription reactions were performed using a TaqMan MicroRNA Reverse Transcription Kit (Applied Biosystems, Foster City, CA) according to the manufacturer’s instructions. Quantitative real-time polymerase chain reaction (qRT-PCR) was performed using the reverse transcription reaction product, TaqMan MicroRNA Assay kit, and TaqMan Universal PCR Master Mix (Applied Biosystems) according to the manufacturer’s instructions. TaqMan MicroRNA Assay kits for human miRs were used. Reactions were loaded onto a 96-well plate and run in duplicate on an ABI 7500 Fast Real-Time PCR System (Applied Biosystems). The reactions were incubated at 50°C for 20 seconds and 95°C for 10 minutes, followed by 40 cycles of denaturation at 95°C for 15 seconds, then 1 minute of annealing/extension at 60°C. The ΔΔC_T_ method was used to determine relative number of copies (RQ) of miR. Data were normalized to a *C*. *elegans* synthetic miR sequence, cel-miR-39 (Qiagen), which was spiked in as a control during RNA isolation.

### Circulating angiogenic proteins

Plasma VEGF, IL-8, and bFGF levels were measured using enzyme linked immunosorbent assay kits (R&D Systems, Minneapolis, MN) according to the instructions of the manufacturer. Results are expressed as pg/ml.

### CEC enumeration

The CellTracks® AutoPrep® System and the CellSpotter® Analyzer II System (Veridex, LLC, Raritan, NJ) were used to enumerate CEC, as has been described [21]. In brief, 4 ml of blood were used for immunomagnetic enrichment using ferrofluids coupled to an anti-CD146 antibody. This marker is present on endothelial cells, a subset of activated T-lymphocytes, and melanoma cells. After enrichment, the following reagents were added: the nuclear dye 4,6- diamidino-2-phenylindole (DAPI), and fluorochrome-conjugated monoclonal antibodies: phycoerythrin-conjugated CD105, which is present on endothelial cells, activated monocytes, and pre-B-lymphocytes, and allophycocyanin-conjugated CD45, a pan-leukocyte antigen included in order to exclude hematopoietic cells from analysis. Analysis was done using image cytometry, where CEC were defined as being CD146+, DAPI+, CD105+ and CD45–. Results are expressed as number of CEC per 4 ml blood.

### Statistical analysis

Data are presented as means ± SEM. Differences were evaluated using unpaired *t* tests, whereas variations between pre- and post-treatment samples were analyzed using paired *t* tests. Spearman rank correlations were used to summarize overall relationships. *P* <0.05 was considered significant. All tests of statistical significance were 2-sided and performed using SAS version 9.2 (SAS Institute Inc, Cary, NC).

## Results

### Angioregulatory miRs

**Figure 1:**
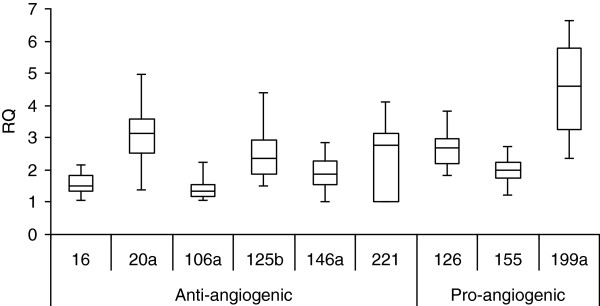
**Box plots of anti- and pro-angiogenic miR levels prior to starting treatment, n = 21.** Horizontal lines represent the median, the box represents the 25th and 75th percentiles, and whiskers represent the minimum and maximum.

**Figure 2:**
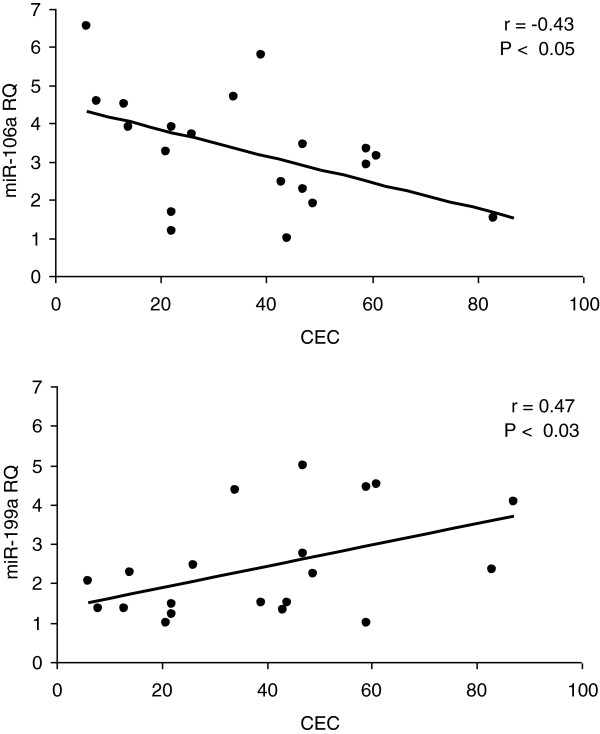
Correlations between miR-106a (top) and miR-199a (bottom) levels and CEC prior to starting treatment, n = 21.

**Figure 3:**
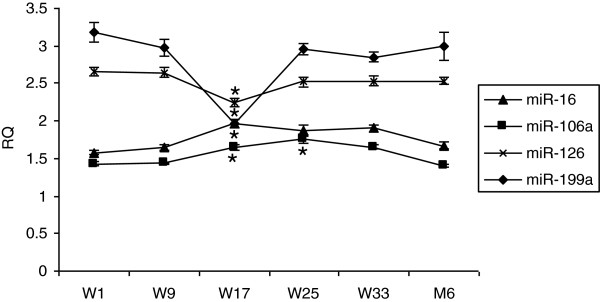
**Levels of miR-16, 106a, 126, and 199a of patients administered sequential dacarbazine and interferon-alfa-2b prior to starting treatment (W1), at weeks (W) 9, 17, 25, 33, and at 6 months (M6) after completing treatments.** Data represent means ± SEM; n = 12. * = *P* <0.05 compared to W1.

Blood was collected from patients with uveal melanoma enrolled on an adjuvant therapy trial in which they were treated sequentially with dacarbazine and interferon. All patients had undergone primary therapy. All patients tolerated treatment, and all completed the program without delays or dose modifications. All patients were disease-free when evaluated 6 months after completion. Plasma levels of nine miRs implicated in regulating angiogenesis were quantified using qRT-PCR. All nine evaluated were measurable in plasma week 1, prior to starting therapy (Figure 1). Levels of pro-angiogenic miRs did not differ from anti-angiogenic. Levels of miR-199a were positively correlated and levels of miR-106a were negatively correlated with CEC levels pre-therapy (Figure 2). A negative correlation of miR-221 (r = -0.40) with CEC did not reach the level of significance (*P* < 0.09). None of the nine miRs tested were correlated with angiogenic protein levels. Plasma levels of miRs did not change at week 9, after two infusions of dacarbazine. At week 17, after eight week of interferon-alfa-2b, levels of miR-126 and 199a decreased, and levels of miR-16 and 106a increased (Figure 3). The increase in miR-106a persisted at day 25; changes in miR-16, 126, and 199a, did not. At week 33 and at 6 months, levels of these miRs returned to baseline. The correlation of levels of miR-199a and 106a with CEC observed at baseline did not persist during therapy, r = -0.28 and r = −0.24, respectively. Significant changes in the levels of miR-20a, 125b, 146a, 155, and 221 were not observed at any time point. Levels of these miRs during therapy also did not correlate with CEC.

### Angiogenic proteins and CEC

**Figure 4:**
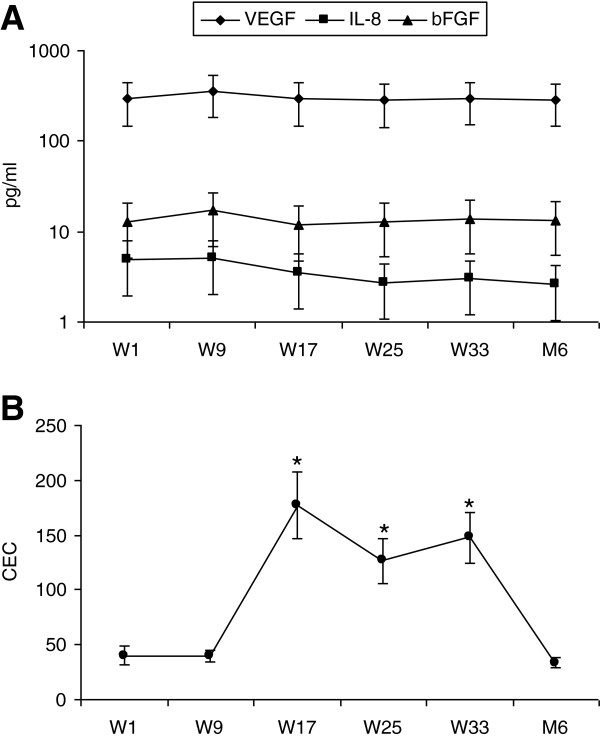
**(A) Angiogenic protein levels and (B) CEC of patients administered sequential dacarbazine and interferon-alfa-2b prior to starting (W1) and at weeks (W) 9, 17, 25, 33 and 6 months (M6) after completing treatments.** Data represent means ± SEM; n = 12. * = *P* <0.05 compared to W1.

VEGF, bFGF, and IL-8 levels were at the same time points that miR levels were determined. Although increases after dacarbazine and decreases after interferon-alfa-2b were observed in individual patients, significant changes in these angiogenic proteins were not apparent at any time point (Figure 4A). From 0 to 97 CEC (mean ± SD = 21 ± 18, median = 15) are found in healthy volunteers, as quantified with the semi-automated immunomagnetic technique used [22]. CEC at week 1, prior to starting therapy, were within this range in all patients. CEC did not change at week 9, after two infusions of dacarbazine. At week 17, after eight week of interferon-alfa-2b, CEC increased significantly. These increases were maintained at week 25, after 16 weeks of interferon, and at week 33, after 24 weeks of interferon. The decreases at weeks 25 and 33 compared to week 17 did not reach the level of significance. After being off treatment for 6 months, CEC levels returned to baseline (Figure 4B). Levels of CEC and angiogenic proteins did not correlate prior, during, or after therapy.

## Discussion

Blood markers are needed to help guide anti-angiogenic therapy. miRs are emerging as important biomarkers, and several miRs have been implicated in regulating tumor angiogenesis [14]. Whether measurement of these miRs in the circulation may be useful clinically has not been established. We found that plasma levels of specific angioregulatory miRs may have utility. There was a positive correlation between levels of the pro-angiogenic miR-199a and a negative correlation between the anti-angiogenic miR-106a with CEC, both moderate, in patients with uveal melanoma prior to receiving systemic adjuvant therapy. miR-199a promotes the proliferation of endothelial cells though effects on caveolin-2 [23]. miR-106a, a paralog of miRs of the miR-17-92 cluster, is upregulated during hypoxia and is predicted to target VEGF [24,25]. With treatment with sequential dacarbazine and interferon-alfa-2b, drugs with anti-angiogenic activities, decreases in miR-199a and in another pro-angiogenic miR, miR-126, and increases in miR-106 and another anti-angiogenic miR, miR-16, were observed. miR-126, an endothelial cell-restricted miR, regulates vascular integrity and angiogenesis. It enhances the actions of VEGF and bFGF by repressing the Spred-1, an inhibitor of angiogenic signaling [26]. miR-16 is implicated in suppressing VEGF and bFGF [27]. miR-16 and 199a have been shown to be produced by human endothelial cells [28]. miR-106a and 199a, as well as miR-146, were among seven miRs found to be increased in the neo-vascularization response to ischemia within the eye in a mouse model [29].

Correlations and changes were not observed in other angioregulatory miRs. These included miR-20a, a pro-angiogenic miR in the 17–92 cluster that represses thrombospondin-1 and connective tissue growth factor [30]; miR-155, which is pro-angiogenic through effects on angiotensin signaling [31], miR-125b, which is anti-angiogenic through effects on placenta growth factor [32]; miR-146a, which is anti-angiogenic though effects of NFκB and suppression of IL-8 and epidermal growth factor receptor signaling [33]; and miR-221, which impairs stem-cell-factor-induced angiogenesis [34]. miR-20a and 221 have also been shown to be produced by human endothelial cells [28]. The relationship between a miR and a target is not exclusive, and depending on what genes are suppressed, a given miR can have either a positive or negative role in the regulation of angiogenesis. Although a member of the pro-angiogenic miR-17-92 cluster, miR-20a can also suppress angiogenesis by suppressing VEGF [25]. miR-199a also affects hypoxia-inducible factor 1 [35] and is predicted to target VEGF [25]. Furthermore, all the miRs tested target multiple genes not directly or indirectly involved in angiogenesis, and several miRs, such as miR-20a and 106a, have been reported to be upregulated in uveal melanoma [36].

Correlations were not observed between levels of miRs and levels of VEGF, bFGF, and IL-8, angiogenic proteins that have been shown to be produced by uveal melanoma [37]. No patient had clinical evidence of cancer at any time during the assessments. In lung cancer, tumor miR-20 was significantly associated with tumor VEGF, and tumor miR-155, with tumor bFGF [38,39]. We also did not observe consistent effects of treatment with dacarbazine or interferon-alfa-2b on the levels of angiogenic proteins. Although changes in the blood levels of angiogenic proteins have been observed in patients administered a variety of anti-angiogenic treatments, they have not been consistent [1]. In one study in patients with cutaneous melanoma administered interferon-alfa-2b, IL-8 levels increased while VEGF and bFGF did not change [40]; in another, levels of VEGF decreased, while levels of bFGF and IL-8 did not change [41].

Significant changes in levels of miRs and also of CEC were observed after treatment with interferon-alfa-2b, but not after treatment with dacarbazine. In contrast to interferon-alfa-2b, the dose and scheduling of dacarbazine were likely not optimal to demonstrate angioregulatory effects, more usually achieved with more repetitive, *i*.*e*., “metronomic,” dosing [17]. The increases in CEC were most apparent when assayed at the week 17 time point, after 8 weeks of interferon, and persisted. The increases in pro-angiogenic and the decreases in anti-angiogenic miRs observed also were most apparent at week 17, but were transient. Interferon-α, which has immune modulatory, antiproliferative, and antiviral effects, has been shown to increase specific miRs by melanoma cells [42]. Whether angioregulatory miRs are modulated has not been reported. Increases in several miRs were observed in patients with chronic hepatitis C virus infection treated with pegylated interferon-alfa-2b but did not correlate with viral load or liver function tests [43].

Whether blood levels of the miRs studied as well other miRs may be useful in monitoring anti-angiogenic therapy merits further investigation. The relative prognostic and predictive value of blood miRs compared to CEC, measurement of which has yielded conflicting results, also merits further evaluation. Of note, levels of angioregulatory miRs during treatment were not significantly correlated with CEC. The clinical situation, the anti-angiogenic approach, and the molecular targets will need to be considered. Given the complexity of the angiogenic process, a combination of several types of biomarkers may be necessary. Studies wherein miR levels and CEC are being assessed in conjunction with liver function tests and imaging studies as part of systemic surveillance for metastases in patients with uveal melanoma are underway.

## Conclusions

Measuring blood levels of specific miRs implicated in angiogenesis, including miR-16, 106a, 126, and 199a, may have clinical utility in monitoring anti-angiogenic therapy in patients with cancer.

## Abbreviations

bFGF: Basic fibroblast growth factors; CEC: Circulating endothelial cells; DAPI: 4,6-diamidino-2-phenylindole; IL-: Interleukin-; miR: MicroRNA; qRT-PCR: Quantitative real-time polymerase chain reaction; RQ: Relative number of copies; VEGF: Vascular endothelial growth factor.

## Competing interests

The authors declare that they have no competing interests.

## Authors’ contributions

PLT conceived and designed the study, supervised its execution, analysis and interpretation, and wrote the manuscript. WA processed the sample, executed the immunoassays, and assisted in their analysis. SA processed the samples, executed the miR studies, and assisted in their analysis. ADS assisted in subject accrual and monitoring. RG executed the circulating endothelial cell studies. ECB helped conceive the study and participated in its design and coordination. All authors read and approved the final manuscript.
